# Evidence of non-response bias in the Press-Ganey patient satisfaction survey

**DOI:** 10.1186/s12913-016-1595-z

**Published:** 2016-08-04

**Authors:** A. R. Tyser, A. M. Abtahi, M. McFadden, A. P. Presson

**Affiliations:** 1 Department of Orthopaedics, University of Utah, 590 Wakara Way, Salt Lake City, UT 84108 USA; 2 Department of Internal Medicine, Division of Epidemiology, University of Utah, 295 Chipeta Way, Salt Lake City, UT 84108 USA

## Abstract

**Background:**

Measures of patient satisfaction have gained prominence in recent years as changes to the American health care system have led to the incorporation of such metrics into reimbursement models. The response rate for widely-used outpatient satisfaction metrics and variables influencing the probability of survey nonresponse remain largely unknown.

**Methods:**

We reviewed all unique adult patients (16,779) who completed an outpatient encounter in the Department of Orthopaedic surgery at our academic institution from 1/1/13 to 10/24/13. Survey data was linked to each clinic visit, and patient factors including age, sex, insurance type, zip code, and orthopaedic subspecialty visited were recorded.

The overall survey response rate was calculated. Logistic regression was performed, and unadjusted and adjusted odds ratios of patients’ probability of responding to the Press-Ganey survey were calculated.

**Results:**

Two thousand seven hundred sixty two (16.5 %) of individuals completed a Press-Ganey patient satisfaction survey and 14017 patients did not respond. For those patients considered responders, 906 patients (32.8 %) did not complete all the survey items. Among these 906 patients, the mean number of missing items was 2.24 (Standard Deviation SD: 2.19).

Age, sex, insurance type, and orthopaedic subspecialty were all found to be associated with the odds of responding to our patient satisfaction survey. Advancing age increased the odds of responding to the survey (Adjusted Odds Ratio (OR) = 3.396 for ≥65 vs. 18–29, *p* < 0.001). Several variables were associated with a decreased odds of survey response, and included male sex (Adjusted OR = 0.782 for Males vs. Females, *p* < 0.001), insurance type (Adjusted OR = 0.311 for Medicaid/Self-Pay vs. Private), and subspecialty type (Adjusted OR = 0.623 for Trauma vs. Adult Reconstruction).

**Conclusions:**

The response rate to the Press-Ganey Medical Practice Survey of outpatient satisfaction is low in an orthopaedic outpatient population, and furthermore, is impacted by patient characteristics such as age, sex, insurance type, and type of orthopaedic subspecialist encountered. The findings of the present study should inform future non-response weighting procedures in this area. More research is needed to assess non-response bias—including follow-up studies of non-respondents—in order to more accurately measure of patient satisfaction.

## Background

Efforts to improve the value of health care in the United States have gained attention in recent years. As a part of that process there has been increasing emphasis on defining and measuring health care quality. Many hospitals and clinics now utilize surveys of the patient experience of care—commonly referred to as patient satisfaction—in the assessment of health care delivery. Recent health care legislation has placed considerable emphasis on the measurement of health care quality and has begun to incorporate metrics of patient satisfaction into reimbursement models. A variety of patient satisfaction surveys are currently being used to make comparisons between clinics, hospitals, and health care systems. The utility and interpretation of these metrics have recently led to substantial debate in both the lay and academic press [1–5].

While reporting of patient satisfaction data has represented a step forward in the ability to measure certain aspects of health care delivery related to the patient experience of care, there are important and well-recognized limitations of such data when used to compare physicians, departments, hospitals, or health care systems [1, 2, 6–8]. In particular, satisfaction survey data is inherently at risk of incurring sampling error, and issues related to effective and equitable use of patient survey data have been noted in several studies on the administration and interpretation of the Hospital Consumer Assessment of Healthcare Providers and Systems (HCAHPS) survey, currently the most utilized measure of patient satisfaction in the United States [9–11]. The results of the HCAHPS survey are both publically reported and tied to funding from the Centers for Medicare and Medicaid Services (CMS) [12]. Recently, there has been an increased emphasis on collection of data on the patient experience of care in a wider variety of settings, including outpatient clinic visits [13].

The Clinician and Group CAHPS (CG-CAHPS) is a measure of patient satisfaction analogous to the HCAHPS, but aimed to assess patient satisfaction with both the outpatient setting and the individual provider(s). The Press-Ganey Medical Practice Survey is similar to the CG-CAHPS, and is approved for use in assessing outpatient satisfaction by the National Quality Forum (NQF)—an organization that, in turn, is sanctioned by CMS to evaluate metrics implemented by the Physician Quality Reporting System (PQRS). Currently, (in contrast to HCAHPS data) reporting of outpatient satisfaction data in any form has not been mandated, but it is expected to be in the future [13]. Less has been published about patient satisfaction with regard to the outpatient experience as compared to the HCAHPS, although there is a growing body of literature on the topic of outpatient satisfaction [14, 15].

There are many types of biases that can occur during the collection of any survey-based data, including patient satisfaction data. With the declining rates of survey response, one of these potential sources of study error that has garnered recent attention is that of nonresponse bias [10, 11, 14, 16, 17]. Nonresponse bias occurs when a portion of the surveyed population does not respond to the survey, and furthermore, if there are differences between those who did and did not respond with regard to the variable of interest [16]. This concept differs from the response rate to a survey, which in many cases is used as a surrogate measure of the potential for nonresponse bias [18]. Specifically, it has been assumed that surveys with higher response rates can be considered to have a lower risk of nonresponse bias, although this assumption may not be entirely valid [17, 18].

Importantly, while identification and correction of such potential biases in survey research are considered integral to accurate interpretation of HCAHPS (hospital-based) patient satisfaction data, it is not apparent that corrections for variation in such factors are occurring in either the reporting or the interpretation of satisfaction data from other surveys or at the outpatient level [9]. In order to more effectively use the data from outpatient satisfaction metrics such as the Press-Ganey survey to achieve the goal of improving the quality of health care delivery in the clinic setting, it may be necessary to increase our understanding regarding outpatient satisfaction survey response rates and potential nonresponse bias.

The purpose of this study was to determine the effect of patient characteristics on the probability of survey nonresponse for a commonly administered metric of patient satisfaction in an orthopaedic outpatient population.

## Methods

As part of an ongoing quality improvement initiative, our institution has contracted with the Press-Ganey Corporation to measure patient satisfaction in our outpatient population. The Press-Ganey Medical Practice Survey is a commonly used proprietary measure of patient satisfaction, and is made up of 24 questions grouped into 6 sub-domains that assess an individual patient’s rating of different aspects of health care delivery in the outpatient setting - access (4 questions), moving through your visit (2 questions), nurse/assistant (2 questions), care provider (10 questions), personal issues (4 questions), and overall assessment (2 questions). As a proprietary measure, the Medical Practice Survey is not currently freely accessible. It is available, however, by request from the Press Ganey Corporation [19]. Each question offers a numerical response ranging from 1 “very poor” to 5 “very good.” To assess satisfaction after a clinic visit, all patients are contacted automatically by email and asked to complete the Press-Ganey Medical Practice Survey, administered via an online survey. Those patients who do not complete the survey within 5 days are then sent another email. The links to the online survey are live for 30 days. Data were collected by the Press-Ganey Corporation, and reported to our institution, linked to a specific clinic visit.

We retrospectively reviewed all adult patients who completed an outpatient encounter in the Department of Orthopaedic surgery at our academic institution from 1/1/13 to 10/24/13. Data tabulated from each clinic visit included Press-Ganey response or non-response, age, sex, insurance provider, insurance type, zip code, and orthopaedic subspecialty visited. The study population was divided into two groups: “responders” (unique patients who responded to at least one survey) and “non-responders” (unique patients who did not respond to a survey at any point).

The survey response rate was calculated. Among survey responders, responses to individual survey items were also assessed and used to calculate the rate of item non-response among survey responders.

Univariable logistic regression models were used to predict odds of survey response from patient characteristics including sex, age, insurance type, sub-specialty type, and home address distance from the clinic. A multivariable (“adjusted”) model was constructed using all variables. Odds ratios, corresponding 95 % confidence intervals (CIs) and Wald chi-squared test p-values were provided for each variable. Statistical analyses were conducted in SAS v.9.3, significance was evaluated at a 0.05 level, and all tests were two-tailed.

## Results

A total of 16779 patients met criteria for inclusion in this study. Characteristics of the patient population are summarized in Table 1. 2762 individuals completed a Press-Ganey patient satisfaction survey (“responders”), and 14017 patients did not respond (“non-responders”). The survey response rate was 16.5 %. 383 subjects were excluded from the multivariable logistic regression analysis due to missing data on one or more of the predictor variables.

**Table 1:** Study population characteristics

		All subjects
		*N* = 16779
Variable		*N* (%)
Gender	F	8872 (52.9)
	M	7907 (47.1)
Insurance category	Medicaid/Self/Other	1773 (10.6)
	Medicare	3863 (23.0)
	Missing	213 (1.3)
	Private	10,107 (60.2)
	Workers compensation	823 (4.9)
Subspecialty^a^	Foot and ankle	2039 (12.2)
	Hand	2367 (14.1)
	Joints	1240 (7.4)
	Non-operative	5995 (35.7)
	Spine	1080 (6.4)
	Sports	2738 (16.3)
	Trauma	1069 (6.4)
New patient visit	N	7054 (42.0)
	Y	9725 (58.0)
Age		
Mean ± SD		49.8 ± 17.8
Median (IQR)		51 (35–63)
Range		18–99+
Age	18–29	2838 (16.9)
	30–49	5040 (30.0)
	50–64	5086 (30.3)
	> = 65	3815 (22.7)
Distance from Clinic^a^	<50 miles	14,009 (83.5)
	> = 50 miles	2764 (16.5)

^a^Six patients were missing data regarding distance from clinic (all non-responders) and 251 were missing sub-specialty (226 from non-responders, 25 from responders)

Age, sex, insurance type, and orthopaedic subspecialty were all found to be associated with the odds of responding to a patient satisfaction survey, with the findings summarized in Table 2. Advancing age increased the odds of responding to the survey (Adjusted OR = 3.396 for ≥65 vs. 18–29, *p* < 0.001). Several variables were associated with a decreased odds of survey response, and included male sex (Adjusted OR = 0.782 for Males vs. Females, *p* < 0.001), insurance type (Adjusted OR = 0.311 for Medicaid/Self-Pay vs. Private), and subspecialty type (Adjusted OR = 0.623 for Trauma vs. Adult Reconstruction).

**Table 2:** Odds ratios from logistic regression models predicting survey response in univariate (Unadjusted) and multivariate models (Adjusted)^a^

Variable	Comparison	Unadjusted odds ratio^b^	Adjusted odds ratio^a^	Adjusted *P* value
Sex	Female (Reference)	--	--	--
	Male	0.757 (0.697–0.823)^c^	0.782 (0.718–0.852)	<.001
Age	18–29 (Reference)	--	--	--
	30–49	1.693 (1.457–1.967)^c^	1.708 (1.468–1.988)	<.001
	50–64	2.697 (2.334–3.117)^c^	2.715 (2.344–3.146)	<.001
	>= 65	2.247 (1.930–2.617)^c^	3.396 (2.815–4.096)	<.001
Insurance	Private (Reference)			
	Medicaid/Self pay/other	0.291 (0.238–0.354)^c^	0.311 (0.255–0.379)	<.001
	Medicare	0.775 (0.700–0.857)^c^	0.478 (0.413–0.553)	<.001
	Workers comp	0.578 (0.466–0.718)^c^	0.588 (0.472–0.733)	<.001
Subspecialty	Adult reconstruction (Reference)	--		--
	Foot and ankle	0.970 (0.814–1.155)	1.048 (0.876–1.253)	0.6086
	Sports	0.836 (0.706–0.989)^c^	0.981 (0.824–1.168)	0.8282
	Hand	0.707 (0.593–0.844)^c^	0.795 (0.663–0.953)	0.013
	Spine	0.686 (0.554–0.851)^c^	0.755 (0.607–0.940)	0.012
	Non-operative	0.682 (0.585–0.796)^c^	0.757 (0.645–0.888)	<.001
	Trauma	0.504 (0.400–0.635)^c^	0.623 (0.492–0.789)	<.001
Distance	> = 50 miles (Reference)			
	<50 miles vs	1.106 (0.988–1.239)	1.103 (0.980–1.241)	0.104

^a^Due to missing data on at least one of the predictor variables, 251 subjects were excluded, leaving 2737 in the responder group and 13791 in the non-responder group

^b^These values are given as the odds ratio (OR), with the 95 % confidence-interval (CI) in parentheses, predicting survey response in univariate (unadjusted) and multivariate (adjusted) models, adjusting for gender, age, insurance type, sub-specialty and distance from the clinic

^c^Significant at the 0.05 level in the unadjusted analysis

## Discussion

With the increased emphasis on value-based care and measurements of quality in health care, patient satisfaction metrics have become both increasingly important and increasingly controversial among those in the medical community. On the one hand, there are concerns that measures of patient satisfaction with the care experience may be used as a surrogate for satisfaction with clinical outcome despite conflicting evidence for an association between patient satisfaction and medical outcomes [1, 20–22]. On the other hand, it appears that the utilization of patient satisfaction data is here to stay (as evidenced by recent legislation), and has the potential to help improve the quality of health care delivery, and possibly the care itself [2, 23].

The results of this study demonstrate that the response rate to the Press-Ganey Medical Practice Survey in an outpatient orthopaedic setting is lower than published reported response rates to other patient satisfaction surveys, and therefore the survey results may be more affected by nonresponse bias. Published response rates to CAHPS surveys fall between 34 and 61 % [23]. In the current study, the response rate to the Press-Ganey survey was 16.5 %.

Potential reasons for the lower response rate identified by this study are speculative. The Press-Ganey survey is sent—via email—to every eligible patient after every clinical encounter, whereas the CAHPS is administered to a randomly sampled subset of patients only. Unlike the Press-Ganey survey, the CAHPS protocol does not allow for email or web-based data collection, relying on standard mail, telephone, or a mixed method to obtain patient response rates of 38, 27, and 42 %, respectively [9]. Other investigators have demonstrated lower response rates with email or web-based survey administration, and the modality choice alone may account for the low response rate to the Press-Ganey survey reported by this study [24–26]. It was outside the scope of this study, however, to investigate specific causes of the low response rate to the Press-Ganey survey.

There is debate regarding the impact of low response rates in survey research [9, 17, 27]. It has been assumed that there is an association between decreasing response rates and increasing potential for nonresponse bias, but this relationship has been challenged. For instance, a meta-analysis investigating the effect of nonresponse bias did not identify any relationship between response rate and nonresponse bias [17]. Still, some peer-reviewed journals such as the Journal of the American Medical Association (JAMA) mandate a minimum response rate (60 % in this case) to be considered for publication [16]. A 16.5 % response rate to the Press-Ganey survey illustrates what appears to be an open question regarding if or when the results of a survey lose any or all validity due to a low response rate.

Despite the lack of consensus on the importance of survey response rate and its relationship with nonresponse bias, many authors continue to warn of the clear potential for nonresponse bias to introduce error into survey research. For example, in a recent review on perceived shortcomings of patient satisfaction survey data, Price et al. noted that “it is important to be aware of the possibility of [nonresponse] bias and use available information to adjust results so that nonresponse does not result in biased comparisons” [23]. It is worth noting that the models employed in analyzing and comparing HCAHPS data from different hospitals are able to take into account potential nonresponse bias [23]. It is not clear if any routine correction for nonresponse is occurring in the Press-Ganey survey, despite the very low response rate identified in this study.

In addition to the low response rate, the present study also demonstrates significant differences in several sociodemographic variables between responders and non-responders to the Press-Ganey Medical Practice Survey in an outpatient orthopaedic population. Older, female patients, and those with private health insurance were significantly more likely to respond to the survey. Orthopaedic subspecialty also influenced the response rate, with orthopaedic trauma patients being the least likely to respond to the survey.

While there are a limited number of studies to date that have investigated nonresponse bias to patient satisfaction surveys, this study is consistent with prior reports demonstrating that age, sex, and socioeconomic status influence response to a variety of patient surveys [9, 11, 28, 29]. The propensity to respond to surveys of patient satisfaction is likely multifactorial, and underlying patient characteristics or personality traits (other than general measures such as age or sex, for example) that lead to response (or nonresponse) remain largely speculative and difficult to assess. Importantly, some of the same variables that appear to influence the likelihood of responding to the Press-Ganey survey have, in turn, been shown to influence the likelihood of being more or less satisfied with outpatient orthopaedic care [7–9, 30–33]. These findings provide further support to the concept that adjustments to patient satisfaction data should likely be made in an attempt to account for differences in patient populations and to counteract nonresponse bias [28, 34].

This study has a number of limitations which include the fact that it was conducted in an exclusively orthopaedic population seeking care at an academic tertiary care center, and therefore may not be reliably extrapolated to other patient populations. We did not include race in our analysis as our patient population was predominantly Caucasian. It is difficult to assess the cause of the low response rate reported in this study, and it stands in some contrast to other reported rates of response to inpatient satisfaction surveys. Although this study was not designed to address this question, the mode of survey administration has been demonstrated to affect response rates in other studies of patient satisfaction, and may be a variable affecting the low response rate seen here [35]. In this study the surveys were administered exclusively by email and web-based methods, as opposed to mailings or telephone contact.

Broadly, when any survey is widely used as the basis for an external quality indicator or comparison tool, the importance of utilizing sound and transparent methodologies in the development, the administration, and the interpretation of the survey becomes paramount [36]. Despite fundamental importance, it is not immediately clear what methodological processes are occurring in the development of proprietary satisfaction surveys such as the Press-Ganey survey, or in the identification of nonresponse bias in the collected data. Similarly, it is not clear if any correction for nonresponse bias is being employed in the reporting of this survey, despite the fact that various statistical methods exist for this purpose [34]. Given the noted importance ascribed to the results of the Press-Ganey survey, we find this apparent lack of transparency regarding methodological rigor concerning.

The trend toward increased emphasis on the measurement of health care quality—including patient satisfaction with care—will likely lead to an increased emphasis on our ability to compare patient satisfaction among different hospitals, clinics, and providers. As supported by this study of orthopaedic outpatient satisfaction survey responses, any valid effort to measure patient satisfaction should be transparent, be held to high methodological standards, and make attempts to prevent and correct for the effects of both low response rates and nonresponse bias.

## Conclusions

Improving the quality of health care delivery relies in part on the accurate measurement of patient satisfaction with the experience of care. In this study done in an American academic orthopaedic clinical setting, the Press-Ganey Medical Practice Survey of patient satisfaction demonstrated a very low response rate and exhibited non-response bias. These findings raise concerns regarding the ability of this metric—and the associated methodologies used to collect it—to accurately measure patient satisfaction. As health care models increasingly utilize patient satisfaction metrics to influence consequential policy decisions and reimbursement structures, the appropriate development, collection, and interpretation of measures such as the Press-Ganey Medical Practice Survey should be emphasized.

## Abbreviations

CG-CAHPS, Clinician and Group CAHPS; CMS, Centers for Medicare and Medicaid Services; IRB, Institutional Review Board; NQF, National Quality Forum; PQRS, Physician Quality Reporting System
